# Fine-Scale Phylogenetic Discordance across the House Mouse Genome

**DOI:** 10.1371/journal.pgen.1000729

**Published:** 2009-11-20

**Authors:** Michael A. White, Cécile Ané, Colin N. Dewey, Bret R. Larget, Bret A. Payseur

**Affiliations:** 1 Laboratory of Genetics, University of Wisconsin, Madison, Wisconsin, United States of America; 2 Department of Statistics, University of Wisconsin, Madison, Wisconsin, United States of America; 3 Department of Botany, University of Wisconsin, Madison, Wisconsin, United States of America; 4 Department of Biostatistics, University of Wisconsin, Madison, Wisconsin, United States of America; 5 Department of Medical Informatics, University of Wisconsin, Madison, Wisconsin, United States of America; 6 Department of Computer Sciences, University of Wisconsin, Madison, Wisconsin, United States of America; University of Aarhus, Denmark

## Abstract

Population genetic theory predicts discordance in the true phylogeny of different genomic regions when studying recently diverged species. Despite this expectation, genome-wide discordance in young species groups has rarely been statistically quantified. The house mouse subspecies group provides a model system for examining phylogenetic discordance. House mouse subspecies are recently derived, suggesting that even if there has been a simple tree-like population history, gene trees could disagree with the population history due to incomplete lineage sorting. Subspecies of house mice also hybridize in nature, raising the possibility that recent introgression might lead to additional phylogenetic discordance. Single-locus approaches have revealed support for conflicting topologies, resulting in a subspecies tree often summarized as a polytomy. To analyze phylogenetic histories on a genomic scale, we applied a recently developed method, Bayesian concordance analysis, to dense SNP data from three closely related subspecies of house mice: *Mus musculus musculus*, *M. m. castaneus*, and *M. m. domesticus*. We documented substantial variation in phylogenetic history across the genome. Although each of the three possible topologies was strongly supported by a large number of loci, there was statistical evidence for a primary phylogenetic history in which *M. m. musculus* and *M. m. castaneus* are sister subspecies. These results underscore the importance of measuring phylogenetic discordance in other recently diverged groups using methods such as Bayesian concordance analysis, which are designed for this purpose.

## Author Summary

The phylogenetic history of individual genes can differ strongly from the species history if taxa are recently derived, making inferences of a species history from only a handful of genes especially difficult in these cases. Genome-scale data sets now allow phylogenetic histories to be reconstructed from a large number of genes. Although data sets of this size are becoming more common, few studies have characterized variation in phylogenetic history across whole genomes. We summarize fine scale variation in phylogenetic history across the genome of house mice, a recently derived group of subspecies, using a method that combines phylogenetic uncertainty among gene trees. We document substantial variation in phylogenetic history among 14,081 loci and describe a primary history in the face of this variation. These results support the use of genome-scale datasets and methods that accommodate phylogenetic discordance in attempts to reconstruct the history of closely related groups.

## Introduction

With the advent of new sequencing technologies, the reconstruction of phylogenetic histories on the genomic scale has become feasible. Genomic data offer the potential to resolve phylogenies that have been difficult to reconstruct from a small number of genes [1]–[5]. Although highly resolved phylogenies can sometimes be recovered when data sets are concatenated, such “total evidence” trees may depart from the history of population branching, the “species history” [6],[7]. The measurement and incorporation of gene genealogical discordance into genomic analyses is expected to improve inferences about species history, particularly for recently derived groups [8].

Topological discordance among gene trees is expected under several scenarios [9]. Population subdivision and asymmetric gene flow among ancestral populations [10], as well as introgression between diverged populations, can generate widespread discordance. Ancestral polymorphisms can also segregate, causing some gene trees to disagree with the population tree. The effects of this incomplete lineage sorting are greatest when effective population sizes are high and internodes of the population tree are of short duration [11]–[16]. Consistent with these predictions, substantial phylogenetic discordance has been documented on the genomic scale in a few young species groups. Pollard et al. [17] demonstrated significant variation among 9,405 genes in *Drosophila erecta, D. melanogaster*, and *D. yakuba*. In addition, genomic discordance has been repeatedly observed in analyses of humans, chimpanzees, and gorillas, with a majority of gene trees supporting a human/chimpanzee sister relationship [18]–[25]. Although it is well established that closely related lineages will exhibit substantial genealogical discordance, few studies have quantified phylogenetic discordance across entire genomes (including non-coding regions). Consequently, the extent of variation on this scale remains poorly understood.

The house mouse subspecies group (*Mus musculus musculus*, *M. m. castaneus*, and *M. m. domesticus*) provides an excellent system for exploring genome-wide patterns of phylogenetic discordance because (i) sources of potential discordance (incomplete lineage sorting and introgression) exist and (ii) almost complete genome sequences are available. The earliest divergences in the house mouse subspecies group occurred only 500,000 generations ago (assuming 1 generation per year) [26]–[30] and house mice are estimated to have large effective population sizes (approximately 10^5^) [30],[31], suggesting an important role for incomplete lineage sorting. In addition, the extent of interspecific gene flow varies across the genome and among the three subspecies [30], [32]–[38]. Two of the subspecies (*M. m. domesticus* and *M. m. musculus*) meet in a stable hybrid zone, in which dramatic variation in introgression among genomic regions has been documented [34],[36],[38],[39]. The other two subspecies pairs (*M. m. castaneus*/*M. m. domesticus* and *M. m. musculus/M. m. castaneus*) also exchange genes in nature, as evidenced by the existence of hybrids [40]–[43]. Furthermore, house mice are model systems for the genetics of speciation [44]–[49], providing the potential to connect genomic variation in phylogenetic history to the evolution of reproductive barriers.

Previous phylogenetic analyses of house mouse subspecies have revealed signs of discordance among genomic regions. Analyses of mitochondrial genes [50],[51], and studies of a handful of genes on the Y chromosome [52],[53], the X chromosome [30], and the autosomes [30],[53],[54] have yielded support for a *M. m. musculus*/*M. m. castaneus* sister relationship. In contrast, complete mitochondrial genome sequences have been unable to resolve the branching pattern [55]. As an increasing number of loci and individuals (within subspecies) have been surveyed, greater evidence for conflicting (but individually well-supported) topologies and non-monophyletic clades has surfaced [30],[53],[54].

The discordance observed in these small datasets motivated us to characterize how frequently gene genealogies fluctuate across the genome and whether a primary phylogenetic history can be identified among the discordance. The accuracy of species tree inference is improved more by increasing the number of loci sampled than by increasing the number of individuals sampled at each locus [12]. In this three-taxon case, the primary phylogenetic history should follow the subspecies tree [56]. We applied a recently developed analytical approach designed to measure and incorporate phylogenetic discordance to genome sequences from *M. m. musculus, M. m. castaneus*, and *M. m. domesticus*. We document substantial, fine-scale discordance among genomic regions and report a primary phylogenetic history for house mice supported by a plurality of the genome. We interpret these results in the context of population genetic processes, including speciation, in house mice.

## Results

### Species and Sequence Data

**Figure 1:**
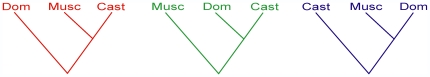
Three possible phylogenetic histories. The three possible phylogenetic relationships among subspecies of house mice are shown, rooted by rat.

To reconstruct the phylogenetic history of house mice, we analyzed genome sequences of three wild-derived inbred strains obtained by Perlegen Sciences using high-density 25-mer oligonucleotide arrays [57]: WSB/EiJ (*M. m. domesticus*), PWD/PhJ (*M. m. musculus*), and CAST/EiJ (*M. m. castaneus*). With three subspecies, there were three possible rooted topologies (Figure 1). We identified assays within the Perlegen data set that had a single nucleotide polymorphism (SNP) between one of the three strains. For each assay, we replaced the C57BL/6J genotype with the strain-specific genotype in the mouse genome [58] using positional information from NCBI build 36, to create a unique genome sequence for each strain. Between the three inbred strains, there were a total of 4,359,927 SNPs genome-wide, with an average of 2.35 SNPs/kb. Estimates from natural populations of the three species of mice have revealed an average divergence among a handful of loci of about 5 SNPs/kb (in all three pairwise species comparisons) [30]. The lower SNP density in this data set reflects efforts by Perlegen to minimize the false positive rate at the expense of a high false negative rate during resequencing [57],[59]. We used the *Rattus norvegicus* genome sequence [58] as an outgroup in all phylogenetic analyses. Rat, which diverged from house mice 12–24 million years ago [60],[61], was the most closely related species with a complete genome sequence available. The entire data matrix contained a total of 1,085,916 parsimony phylogenetic informative sites across the genome, with an average of 604 informative sites per Mb.

### Genome Partitioning

We partitioned the genome for subsequent phylogenetic analyses using the minimum description length (MDL) principle [62], which set breakpoints where shifts in phylogenetic history most likely occurred. In this manner, the genome was partitioned into 14,081 loci with a median size of 98,238 bp and a maximum locus size of 7.21 Mb (Figure S1). Locus size varied widely across the genome (SD 312,637 bp) and was negatively correlated with the density of parsimony phylogenetically informative sites (Spearman's rank correlation, rho  = −0.501, *p*<0.00001), as expected because the genome was partitioned based upon 100 SNP windows rather than windows of constant physical position. In regions of lower SNP density, 100 SNP windows encompassed larger stretches of the genome.

To determine whether the distribution of locus sizes correlated with relevant biological processes (rather than reflecting an arbitrary partitioning based on the density of informative sites), we compared locus size to fine-scale recombination rate across the genome. Three-species models predict that the spatial scale of phylogenetic switching caused by incomplete lineage sorting should be related to the local rate of recombination [63] and consequent scale of linkage disequilibrium in ancestral populations. This pattern has been observed in phylogenomic studies of humans, chimpanzees, and gorillas [24]. We estimated recombination rate in 1 Mb windows using an updated version of the high-density mouse genetic map [64],[65] and used these estimates to examine correlations with locus size across the genomes of the three species. As predicted, we found a low, but significant negative correlation between recombination rate and locus size (Spearman's Rank, n = 14,081, rho  = −0.0632, *p*<0.00001).

Because marker density in the genetic map varies across the genome, the closest estimate of recombination rate can also vary. We repeated the correlation analysis using only loci that were within 100 kb of the nearest recombination rate estimate, resulting in a similar negative correlation (Spearman's Rank, n = 8,344, rho  = −0.0744, *p*<0.00001). We also compared median recombination rates between the longest 2.5% and the shortest 2.5% of the loci. There was a significantly lower median recombination rate in the longer loci (0.292 cM/Mb) as compared to the shorter loci (0.480 cM/Mb; *p*<0.00001) (Figure S2), confirming an effect of recombination on the spatial distribution of phylogenetic discordance.

### Estimating Single-Locus Phylogenetic Histories

**Figure 2:**
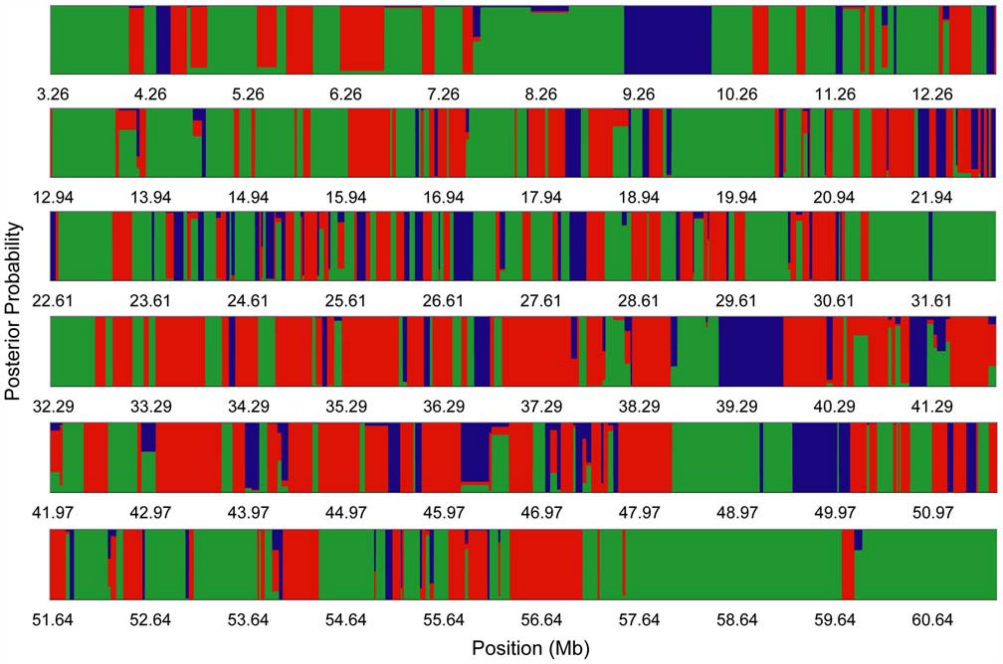
Fine-scale phylogenetic discordance. The posterior probability of each topology is mapped along chromosome 19 to characterize fine-scale patterns of discordance among the 410 loci. Many regions of the chromosome rapidly switch between phylogenetic histories and are characterized by loci that have a high posterior probability for a single topology. Colors correspond to the three topologies.

We separately estimated the rooted gene genealogy of each locus by identifying the best-fitting model of molecular evolution and conducting a Bayesian phylogenetic analysis. These individual loci supported single topologies with high statistical confidence (Table S1). For example, 84.9% of loci supported a single topology with a posterior probability of 0.9 or greater (Figure S3). High posterior probabilities were observed across a wide variation of locus sizes and for each topology (Figure 2; Figure S4). Resolved gene trees can be supported by artificially high Bayesian posterior probabilities if the true tree is a polytomy (the “star tree paradox”) [66]–[69]. To evaluate whether the high support we obtained for each locus was caused by this problem, we calculated the likelihood score of the Bayesian majority rule consensus tree and the likelihood score of the tree with the internal branch constrained to length zero. We computed a likelihood ratio test statistic to determine if the model with an internal branch of non-zero length was a significantly better fit than a model with an internal branch of zero length. Only 247 of the 14,081 loci had internal branches statistically indistinguishable from a tree with a collapsed internal branch (significance set at α = 0.1, 1 df, χ^2^ = 2.706) [70], indicating that the high posterior probabilities we observed generally reflected the accurate resolution of gene trees with short internal branches.

We compared the single-locus posterior probabilities we estimated through the Bayesian phylogenetic analyses with bootstrap support from maximum likelihood analyses to determine if the high support we obtained was due to the choice of methodology. Bootstrap supports from the maximum likelihood analyses were highly correlated with the Bayesian posterior probabilities for each of the three topologies (*M. m. musculus/M. m. castaneus*: Spearman's Rank, n = 14,081, rho = 0.928, *p*<0.00001; *M. m. castaneus/M. m. domesticus*: rho = 0.925, *p*<0.00001; *M. m. musculus/M. m. domesticus*: rho = 0.880, *p*<0.00001), indicating that overall patterns of statistical support at each locus were robust to the method used to reconstruct phylogenetic history.

### Genome-Wide Discordance

**Figure 3:**
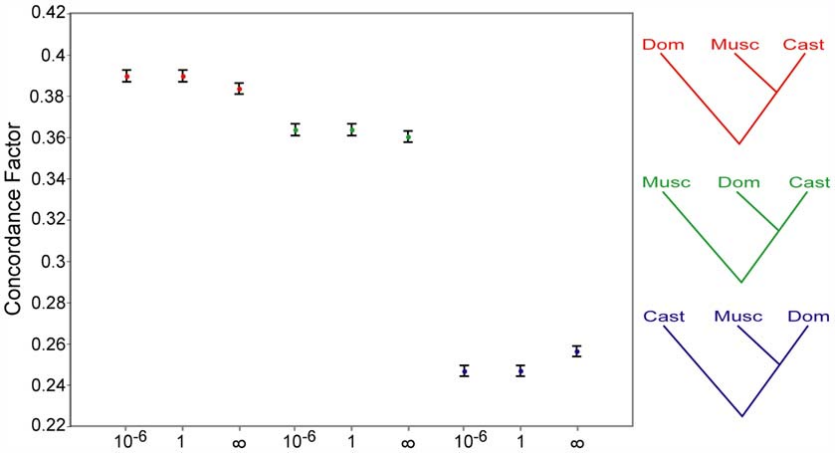
Genome-wide phylogenetic discordance. Bayesian concordance factors were calculated from the posterior probability distributions of 14,081 single-locus phylogenetic analyses. The concordance analysis is depicted using three different priors on gene tree concordance: complete independence among loci (α  =  infinity), a high probability of concordance among loci (α = 10^−6^), and an intermediate level of concordance (α = 1). The *M. m. musculus*/*M. m. castaneus* topology is supported by significantly more loci than the other two topologies regardless of the prior on gene tree concordance. Colors correspond to the three topologies. Error bars are 95% credibility intervals.

To measure phylogenetic discordance across all loci, we used the posterior probability distributions from all single-locus phylogenetic analyses as input for the Bayesian concordance analysis [71]. By incorporating the statistical uncertainty in phylogenetic reconstruction among the individual loci, we were able to estimate the number of loci across the genome that supported each individual topology. Bayesian concordance analysis identified a primary phylogenetic history, placing *M. m. musculus* and *M. m. castaneus* as sister subspecies (Figure 3). This tree was supported by a concordance factor of 0.390±0.003, or 39% of all loci (with a prior probability of gene tree concordance at α = 1). There was also substantial support for the two other possible histories. The *M. m. castaneus*/*M. m. domesticus* clade had a concordance factor of 0.363±0.003, while the *M. m. musculus*/*M. m. domesticus* clade had a lower concordance factor of 0.247±0.003. Although the Bayesian concordance analysis estimates the proportion of loci supporting a particular topology, the analysis does not integrate the sizes of individual loci. If the concordance factors are accurately reflecting the contributions of incomplete lineage sorting and gene flow, the median locus sizes supporting each of the three topologies should parallel the concordance factors. Median locus size followed the same rank order as the concordance factors, with the *M. m. musculus/M. m. castaneus* topology having the largest locus size (Figure S5).

Other researchers have computed concordance factors using maximum likelihood methods by filtering out gene trees with less than 95% bootstrap support and calculating the proportion of loci from the filtered pool that supported each topology [23]. We also repeated our concordance analysis using maximum likelihood methods. 7,260 of the 14,081 loci had at least 95% bootstrap support. Of these trees, *M. m. musculus/M. m. castaneus* was supported by a concordance factor of 0.415, *M. m. castaneus/M. m. domesticus* had a concordance factor of 0.366, and *M. m. musculus/M. m. domesticus* had a concordance factor of 0.219. These concordance factors were similar to those resulting from Bayesian concordance analysis, indicating that our conclusions were robust to alternative analytical approaches. The slight differences likely arose because Bayesian concordance analysis used all loci and incorporated uncertainty across loci, whereas the maximum likelihood method only used half of the available loci and did not propagate uncertainty across loci.

Bayesian concordance analysis uses a prior probability of gene tree concordance, which could affect our estimates of a primary phylogenetic history. To address this issue, we recalculated concordance factors assuming two extreme priors: (1) a high probability of concordance among individual loci, and (2) complete independence among individual loci. In both cases, patterns of discordance among the three histories remained largely unchanged (Figure 3), suggesting robustness to prior assumptions. This robustness was likely due to the large number of loci used in the concordance analysis combined with the high degree of support for single topologies at most individual loci.

Estimates of concordance might also be affected by the parameters used in the MDL partitioning (the cost and the starting interval size). To investigate this possibility, we applied the maximum (3) and minimum (0.9039) costs against splitting concatenated fragments in the MDL partitioning on chromosomes 18, 19, and X. In all cases, partitioning the genome with the minimum cost roughly doubled the number of loci on each chromosome, but chromosome-wide concordance factors were not significantly altered (Figure S6). We also calculated concordance factors on chromosomes 18 and 19 using a range of starting interval sizes (25, 50, 100, 150, 200, and 250 SNPs). For both chromosomes, concordance factors did not significantly differ for the three topologies with starting interval sizes of 25, 50, or 100 SNPs when the full range of credibility intervals were taken into account (Figure S7), indicating that partitioning with starting intervals smaller than 100 SNPs did not significantly alter the estimates of concordance.

The phylogenetic histories at individual loci may not be the true histories if the divergence time between rat and house mice is too great [72],[73]. Under this scenario, the rat branch can pair with whichever mouse lineage has the greatest amount of divergence and the largest number of sequence similarities due to homoplasy rather than orthology (long-branch attraction). To evaluate this possibility, we randomized the nucleotides of the rat sequence at each locus, erasing any phylogenetic signal and further compounding the effect of long-branch attraction. The posterior probability distributions from each shuffled locus on chromosomes 18 and 19 were used as input for the Bayesian concordance analysis [71]. If the patterns we observed in the data were due to long-branch attraction, we would expect to recover similar patterns of discordance with an artificially lengthened branch. Instead, we found a large difference in discordance between the randomized and nonrandomized data sets, with the randomized data set not differing from concordance factors of 1/3 (chromosome 18: χ^2^ = 4.900, df = 2, *p* = 0.086; chromosome 19: χ^2^ = 3.286, df = 2, *p* = 0.193) (Figure S8). This indicated that the rat sequence provided strong phylogenetic signal.

### Patterns of Discordance Within the X Chromosome

**Figure 4:**
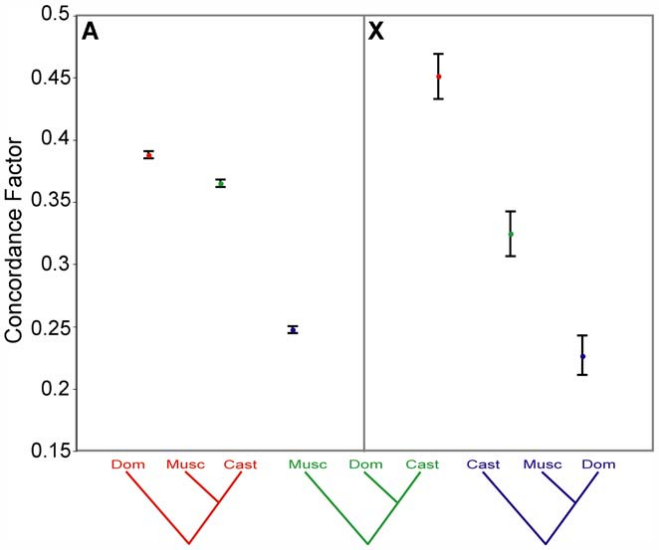
Phylogenetic discordance between the autosomes and the X chromosome. Significantly less discordance is observed across loci of the X chromosome (X: 442 loci) than the autosomes (A: 13,639 loci). This is shown by significantly higher support for the primary phylogenetic history, the *M. m. musculus/M. m. castaneus* topology, at the expense of loci supporting the other two topologies. Colors correspond to the three topologies. Error bars are 95% credibility intervals.

Patterns of phylogenetic discordance within the X chromosome are expected to differ from those on the autosomes. The X chromosome has a smaller effective population size than the autosomes (¾ as large, assuming a breeding sex ratio of one) leading to the prediction that ancestral polymorphism should sort more quickly. Additionally, loci on the X chromosome exhibit reduced gene flow within and between species of house mice [30],[32],[33],[36],[38],[39],[74],[75]. Both factors should reduce discordance across the X chromosome. In agreement with patterns for the autosomes, the primary phylogenetic history of the X chromosome was a *M. m. musculus*/*M. m. castaneus* sister relationship. As predicted, this history was supported by a higher concordance factor (0.450±0.018) on the X chromosome than on the autosomes (Figure 4). In addition, loci supporting a *M. m. castaneu*s/*M. m. domesticus* topology had a lower concordance factor (0.324±0.018) on the X chromosome than on the autosomes. Although the concordance factor supporting a *M. m. musculus*/*M. m. domesticus* topology (0.226±0.016) was also lower than the autosomes, the 95% credibility intervals overlapped. Median locus sizes matching each topology on the X chromosome also paralleled the concordance factors (as observed on the autosomes), with the *M. m. musculus/M. m. castaneus* topology showing a larger deviation in size from the two minor topologies (Figure S5).

Increased support for the *M. m. musculus/M. m. castaneus* primary phylogenetic history and decreased support for the *M. m. castaneus/M. m. domesticus* minor history indicated reduced phylogenetic discordance on the X chromosome. To determine whether the reduced discordance simply arose from differences in sample size (n_X_ = 442 loci, n_autosomes_ = 13,639 loci), we compared the concordance factors on the X chromosome to concordance factors estimated from sets of 442 randomly drawn loci from the autosomes (5000 replicates). The reduced discordance on the X chromosome persisted in these comparisons (*M. m. musculus/M. m. castaneus*: higher, *p* = 0.0062; *M. m. castaneus/M. m. domesticus*: lower, *p* = 0.038; *M. m. musculus/M. m. domesticus*: no difference, *p* = 0.326). Reduced discordance on the X chromosome thus appears to reflect processes differentially affecting the X chromosome and the autosomes.

### Ascertainment Bias in SNP Discovery

**Figure 5:**
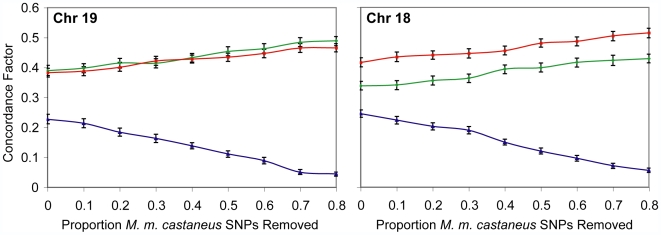
Simulated ascertainment bias against *M. m. castaneus*. Varying proportions of *M. m. castaneus* specific SNPs were removed from chromosomes 18 and 19 to simulate increased ascertainment bias against this taxon. The difference in concordance factors between *M. m. musculus*/*M. m. castaneus* and *M. m. castaneus*/*M. m. domesticus* do not significantly differ at all levels of artificial bias. This indicates that recovery of a *M. m. musculus/M. m. castaneus* primary phylogenetic history is robust to increased ascertainment bias. Colors correspond to the three topologies. Error bars are 95% confidence intervals.

A potential source of discordance in our results comes from ascertainment bias in SNP identification. Sequences for the three house mouse species were obtained using arrays designed from a C57BL/6J reference sequence, which assayed for SNPs from the reference sequence by hybridizing oligonucleotide probes. Strong sequence divergence from the reference sequence could result in inefficient hybridization of the probes, increasing the false negative rate by incorrectly calling a C57BL/6J genotype. Consistent with such a bias, a deficiency of SNPs from the three species was documented [59]. The strongest bias was against *M. m. castaneus* specific SNPs – SNPs at which the *M. m. castaneus* strain differs from the *M. m. domesticus* and *M. m. musculus* strain. This deficiency could reduce the number of loci supporting a *M. m. musculus*/*M. m. domesticus* sister relationship (consistent with our results). To determine whether ascertainment bias would affect our ability to resolve a primary phylogenetic history, we simulated increased ascertainment bias against *M. m. castaneus*. *M. m. castaneus* informative SNPs were randomly removed from each locus across chromosomes 18 and 19 at varying levels of severity (ranging from 10–80% removed) by converting the *M. m. castaneus* specific SNP to the C57BL/6J genotype. All phylogenetic analyses were then repeated. These simulations modeled the effects of artificially increasing the false negative rate of SNP identification against *M. m. castaneus*. For each chromosome, as ascertainment bias against *M. m. castaneus* was increased, the concordance factor supporting the *M. m. musculus/M. m. domesticus* topology decreased linearly (Figure 5). Importantly, introducing ascertainment bias did not *differentially* affect the inferred concordance factors for the other two topologies; both factors increased at equal rates. Although ascertainment bias against *M. m. castaneus* SNPs existed in this data set, our main conclusions of a *M. m. musculus*/*M. m. castaneus* primary phylogenetic history and substantial phylogenetic discordance across the genome were mostly unaffected by this bias.

A second form of bias could also increase the branch length of *M. m. domesticus* in relation to the *M. m. musculus* and *M. m. castaneus* branches. Perlegen discarded SNPs that were polymorphic in only one of the 15 strains sequenced. As a majority of the strains were *M. m. domesticus* in origin [57],[59], the analysis favored SNP discovery in this subspecies. If this bias increased the branch length of *M. m. domesticus* across the genome, this could raise support for the primary *M. m. musculus/M. m. castaneus* topology through long-branch attraction to rat. When the rat sequence was randomized, support for the *M. m. musculus*/*M. m. castaneus* and *M. m. castaneus/M. m. domesticus* topologies both decreased. However, on both chromosomes, the *M. m. musculus/M. m. castaneus* topology decreased to a greater degree (Figure S8). This result indicated that the *M. m. musculus/M. m. castaneus* topology was least affected by long-branch attraction and that the high support we observed for the primary phylogenetic history came from orthology to the rat sequence rather than ascertainment bias.

## Discussion

Our genomic analysis revealed a primary phylogenetic history across the house mouse genome, placing *M. m. musculus* and *M. m. castaneus* as sister subspecies. We also documented striking phylogenetic discordance on a genome-wide scale. Discordance was observed in previous phylogenetic studies of house mice based on a small number of loci [30],[53],[54]. In addition, gene trees reconstructed from large population samples have shown that reciprocal monophyly between subspecies is higher on the X chromosome than the autosomes [29]–[31], a result that agrees with our genomic comparisons. In summary, our results extend previous observations from phylogenetic analyses of a few loci to the entire genome, thereby providing the power needed to resolve the history of these closely related subspecies for the first time.

In the case of three taxa where there is an underlying tree-like population history (the “species tree”), the primary phylogenetic history is expected to match the species history, whereas the frequencies of the minor trees should reflect the contributions of incomplete lineage sorting and differential gene flow [9],[10],[12],[56],[76]. Several additional factors might shape the discordance we observed. We now discuss the importance of each potential source of discordance in turn.

### Biases in Phylogenetic Reconstruction

Errors at several stages of phylogenetic reconstruction could generate phylogenetic discordance [76]–[78]. First, mis-estimated models of molecular evolution could introduce disagreement among loci. However, by statistically selecting the best-fitting model of molecular evolution separately for each locus, we minimized errors associated with assuming the same model across loci. Second, the alignment with rat might have inflated discordance if the error rate in the whole-genome alignment was high. Contrary to this idea, randomizing the rat sequence in respect to the three mouse sequences across chromosomes 18 and 19 strongly reduced the posterior probabilities at individual loci and instead exacerbated discordance, suggesting that the rat sequence contributed a strong phylogenetic signal. Third, estimates of concordance might be affected by the parameters used in the MDL partitioning. Applying the minimum cost against splitting concatenated fragments roughly doubled the number of loci, but concordance factors were not significantly altered. In addition, partitioning the genome using starting intervals less than 100 SNPs had no significant effect on the concordance factors. Finally, concordance factors might have been inaccurately estimated because many SNPs were missed by resequencing [57]. Although comparable analyses of complete genome sequences would likely reveal variation in the exact breakpoints of partitions, reduced numbers of informative sites did not seem to be responsible for the observed discordance. Our analyses demonstrated that MDL partitions the genome in a phylogenetically informative manner and that individual loci generally favor one history with high posterior probability. In addition, we found a significant correlation between locus size and recombination rate across the genome (as predicted by theory), suggesting that this dataset contains information about the evolutionary processes responsible for phylogenetic discordance.

Although we detected a significant negative correlation between locus size and recombination rate, the correlation coefficient was relatively low, indicating that most of the variation in locus size was explained by other variables. The weakness of this correlation was expected for several reasons. First, our data set was limited by the number of informative sites generated by the resequencing project. Additional sequence data might change the locations of breakpoints inferred by the MDL partitioning, which would alter the locus sizes and the correlation with recombination rate. Second, the recombination rate estimates came from crosses between other inbred strains of mice [64],[65], not the wild-derived strains used in our analyses. Differences in recombination rate between some of the strains used to construct the mouse genetic map and one of the wild-derived strains included in our analysis (CAST/EiJ) have been observed [79].

### Patterns of Discordance across the House Mouse Genome

Pairwise divergence times between house mouse subspecies pairs are roughly similar when the full range of confidence intervals is considered [30], suggesting a rapid, sequential splitting of the three subspecies. This scenario is expected to result in concordance factors that differ only slightly from 0.333, due to the short internal branch of the phylogeny. Our results are consistent with these patterns, with a primary phylogenetic history supported by a concordance factor of 0.390 across the autosomes. In contrast, three-taxon cases in *Drosophila* and primates feature phylogenies with longer internal branches, resulting in a greater proportion of the genome supporting the primary phylogenetic histories [17],[22],[23],[25].

If incomplete lineage sorting is solely responsible for phylogenetic discordance, the two minor topologies should occur at equal frequencies in the genome [9],[12],[56],[76], and these frequencies should decrease at equal rates as effective population size decreases and the length of the internal branch increases [11],[15]. In contrast, our analysis revealed asymmetric genomic proportions supporting the two minor topologies, indicating a strong deviation from the model of pure lineage sorting. Similar patterns were observed in *Drosophila* species [17], and on the X chromosome in primates [10],[22].

Gene flow following divergence can drive asymmetries between the minor histories. Patterns of shared polymorphism among populations [29]–[31] and introgression across hybrid zones [30], [32]–[39],[74],[75] indicate that gene flow differs among the subspecies pairs and across the genome. If the primary phylogenetic history (*M. m. musculus/M. m. castaneus*) represents the subspecies history, high levels of gene flow from the outgroup (*M. m. domesticus*) into *M. m. castaneus* or *M. m. musculus* could raise support for the minor histories. Significant levels of gene flow have only been detected between *M. m. musculus* and *M. m. castaneus* and between *M. m. domesticus* and *M. m. castaneus* [30]. This introgression is expected to increase support for the *M. m. castaneus/M. m. domesticus* minor history in respect to the *M. m. musculus/M. m. domesticus* minor history, as observed in our data.

In addition to gene flow in nature, sequencing error likely contributed to differences in concordance factors between the two minor histories. It has been suggested that sequencing errors could have caused differences in the genomic proportions supporting alternative minor histories in *Drosophila* and on the primate X chromosome [10]. Resequencing studies have detected a high false negative rate against *M. m. castaneus* specific SNPs in this data set [59]. This bias probably led us to underestimate the concordance factor for the *M. m. musculus/M. m. domesticus* topology. Although we cannot separate the contributions of recent gene flow and ascertainment bias to the asymmetry between minor histories in our analyses, ascertainment bias seems to have played a larger role in producing this pattern. If the asymmetry between minor histories was mostly due to gene flow, we would expect it to be less apparent on the X chromosome because recent introgression has been relatively reduced on the X chromosome [30], [34]–[36],[39],[74],[75]. In contrast, differences between minor histories were similar for the X chromosome (0.099) and the autosomes (0.117) when the full range of credibility intervals was considered, suggesting that gene flow was not the primary underlying cause. Furthermore, the asymmetry was still present after the rat sequence was randomized at each locus across chromosomes 18 and 19. Because the shuffling erased phylogenetic signal due to orthology, lowered support for the *M. m. musculus/M. m. domesticus* topology was apparently caused by a shorter *M. m. castaneus* branch. This result also supports the idea that ascertainment bias contributed to the difference in concordance factors between the two minor histories.

Although ascertainment bias appears to have affected the relative frequencies of the minor histories, it does not seem to have interfered with our identification of a primary phylogenetic history. Randomly removing *M. m. castaneus* informative sites in our simulation study did not alter the difference between the *M. m. musculus/M. m. castaneus* and *M. m. castaneus/M. m. domesticus* topologies; both decreased at equal and linear rates as *M. m. castaneus* informative sites were removed. Assuming the asymmetry was entirely driven by ascertainment bias, we adjusted the data according to the simulations by lowering the concordance factors of the *M. m. musculus/M. m. castaneus* and *M. m. castaneus/M. m. domesticus* topologies equally until the two minor histories had equal concordance factors (*M. m. musculus/M. m. castaneus*: 0.349; *M. m. castaneus/M. m. domesticus*: 0.326; *M. m. musculus/M. m. domesticus*: 0.326). Although these concordance factors do not include any effect of gene flow (an unrealistic assumption), these rough estimates allowed us to calculate the length of the internal branch of the subspecies tree that would maximize the likelihood of our dataset under a model of pure lineage sorting [80]. Using an ancestral population size of 120,000 (an average value across the three pairwise subspecies comparisons) [30] and assuming one generation per year, the concordance factors are consistent with an internal branch length of only 5,520 generations (95% CI: 2,640–8,400 generations). The high level of phylogenetic discordance we observed suggests a rapid splitting of the three house mouse subspecies, consistent with close divergence times among the three subspecies estimated from large population samples [30].

### A Phylogenetic History for House Mouse Subspecies

The *M. m. musculus*/*M. m. castaneus* primary phylogenetic history has significantly higher support across the genome, indicating that it is an accurate reflection of the subspecies tree. Several additional lines of evidence support this conclusion. First, there is significantly higher support for this topology on the X chromosome (relative to the autosomes) where incomplete lineage sorting is expected to be reduced. If either of the minor histories were the true subspecies tree, rates of gene flow would need to be higher on the X chromosome than on the autosomes to explain the difference in concordance factors. However, gene flow on the X chromosome is considerably lower [30],[32],[33],[36],[38],[39],[74],[75].

**Figure 6:**
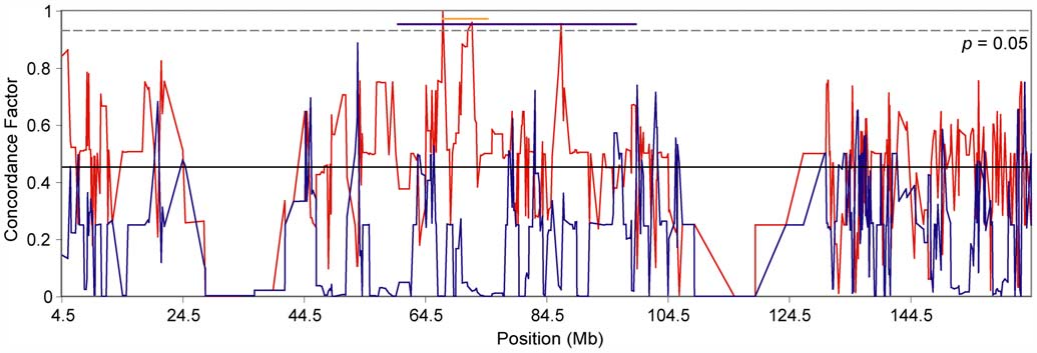
Sliding window analysis of discordance across the X chromosome. Discordance within a four-locus sliding window was calculated across the X chromosome and is plotted as the midpoint position of each window. The *M. m. musculus*/*M. m. castaneus* topology (red) shows significantly higher regions along the X chromosome where known hybrid male sterility loci are mapped (*Hstx1*) [46]. The *M. m. musculus/M. m. domesticus* topology (blue) decreases within these regions. The entire *Hstx1* interval is indicated by the purple line, whereas the peak of this quantitative trait locus is indicated by the orange line. The black line indicates the chromosome-wide concordance factor for the *M. m. musculus/M. m. castaneus* topology (0.450).

Second, we observed increased support for the *M. m. musculus*/*M. m. castaneus* primary history at a hybrid male sterility locus. In species that experience gene flow after the initial development of reproductive isolation, loci underlying reproductive barriers might better reflect species history because discordance generated by gene flow is reduced in these regions [76],[81]. Increased phylogenetic resolution of species history has been observed at loci associated with hybrid male sterility [82]–[84]. Within house mice, loci that affect hybrid male sterility have been mapped repeatedly to the X chromosome in crosses between *M. m. musculus* and *M. m. domesticus* [46],[48],[49]. As a preliminary examination of the association between reproductive isolation loci and phylogenetic history, we performed concordance analyses in sliding windows comprised of four contiguous loci across the X chromosome. We identified several adjacent regions supporting a *M. m. musculus/M. m. castaneus* topology with significantly higher concordance factors than the remainder of the X chromosome (*p*<0.05; calculated by comparison to results using random subsets of four loci; Figure 6). The highest peak spanned a 1.55 Mb region and was supported by a concordance factor of 0.998 (*p* = 0.0196). This region matched the estimated location of the hybrid male sterility locus identified by Storchová et al. [46]. As might be predicted from the mapping results, concordance factors supporting the *M. m. musculus*/*M. m. domesticus* topology were also markedly reduced in these regions. We did not detect similar associations for other known hybrid sterility loci, including *Hst1* on chromosome 17 [85]. However, the results from the X chromosome should motivate similar analyses across the entire genome once more information is available about the regions contributing to reproductive isolation in house mice.

In addition to informing speciation studies, the phylogenetic history of house mouse subspecies has important implications for mouse genetics. The classical inbred mouse strains widely used in genetic studies of disease and other phenotypes are descended – in unequal proportions – from the three subspecies examined here [86],[87]. Analyses of the Perlegen sequences documented substantial genomic variation in relationships among the classical strains and attempted to attribute the ancestry of different genomic regions to *M. m. domesticus*, *M. m. musculus*, and *M. m. castaneus* [57],[59]. Our results suggest that much of this phylogenetic variation likely reflects incomplete lineage sorting and differential introgression in wild mice. Genomic comparisons involving larger numbers of wild mice will be needed to interpret the patterns of genetic variation observed in the classical strains.

## Methods

### Sequence Data

Phylogenetic analyses were conducted using genome sequences from three wild-derived inbred strains: WSB/EiJ (*M. m. domesticus*; Maryland), PWD/PhJ (*M. m. musculus*; Czech Republic), and CAST/EiJ (*M. m. castaneus*; Thailand). Although WSB originated in North America, *M. m. domesticus* from eastern North America are genetically similar to European *M. m. domesticus* that are closer to the ancestral subspecies range [88]. For each strain, a genome sequence was reconstructed using single nucleotide polymorphism (SNP) resequencing data generated by Perlegen Sciences [57]. This data set was obtained by using an oligonucleotide array designed with the C57BL/6J strain as a reference sequence. We identified 4,359,927 million assays within this dataset that were polymorphic in one of the three strains (WSB/EiJ, PWD/PhJ, or CAST/EiJ). For each assay, we replaced the C57BL/6J genotype with the strain-specific genotype in the mouse genome [58] using positional information from NCBI build 36. Any missing SNP data (N's) were also substituted in to the subspecies-specific genome sequence. The completed genome sequences consisted of SNPs at an average density of 2.35 SNPs/kb, surrounded by large tracks of C57BL/6J genome sequence, which were invariant between the three subspecies.

### Whole-Genome Alignment

To root the phylogenetic analyses, the genome sequence from *Rattus norvegicus* [60] was used as an outgroup. Rat was chosen because it was the most closely related species to mouse with a whole-genome sequence available. The entire C57BL/6J genome was used in the alignment except for chromosome Y, as there is no rat chromosome Y sequence available. The NCBI build 36 mouse genome sequence was aligned to version 3.4 of the rat genome using a combination of the Mercator and MAVID programs [89]. Mercator was used to build a one-to-one colinear orthology map between the two genomes and MAVID was run on the resulting colinear blocks to produce nucleotide-level alignments. The input to Mercator consisted of all coding exon annotations for mouse and rat available from the UCSC Genome Browser [90] as well as the results from running BLAT [91] on the coding exon sequences in an all-vs-all fashion. Although coding regions formed the basis for the whole-genome alignment, all sequence data (coding and non-coding sequences) were utilized in subsequent phylogenetic analyses.

### Minimum Description-Length Genome Partitioning

Orthologous blocks created from the alignment with rat were further partitioned into loci by measuring phylogenetic incongruence within the blocks using a minimum description length (MDL) principle [62]. Each orthologous block was split into consecutive 100 SNP intervals. Partitions were formed by combining these intervals into longer fragments. The Description Length (DL) of a partition was determined by the sum of the maximum parsimony tree lengths of each fragment in the partition, plus a penalty for the number of fragments. The penalty was set to be a constant cost (C) times the number of fragments. The partition with the smallest DL was selected. For example, consider the comparison between a partition with two adjacent fragments and a partition with the two fragments concatenated into a single large fragment. The penalty was 2*C for the former partition and 1*C for the latter. Concatenation was favored when the difference between the tree length of the two concatenated fragments and the sum of the tree lengths of the non-concatenated fragments was smaller than C (the penalty difference). Alternatively, two adjacent fragments were kept separate if the difference was greater than C, reflecting a shift in phylogenetic history of one region. The maximum and minimum costs for four taxa were used. The maximum cost favored fewer breakpoints whereas the minimum cost favored more breakpoints. A maximum cost of three was based on equation (2) in Ané and Sanderson [62]. A lower cost of 0.9039 was also used. A range of starting interval sizes was used (25, 50, 100, 150, 200, and 250 SNPs) on chromosomes 18 and 19 to determine the largest starting interval size that had similar concordance factors as the smaller interval sizes. This ensured the highest possible resolution while maintaining a reasonable computational time.

### Single-Locus Phylogenetic Analyses and Bayesian Concordance Analysis

Each locus identified from the MDL partitioning was subjected to a separate Bayesian phylogenetic analysis. Each locus was allowed to follow an independent model of molecular evolution, as determined by MrModelTest (Nylander 2004; http://www.abc.se/~nylander/mrmodeltest2/mrmodeltest2.html). The highest scoring model was selected based upon Akaike's information criterion (AIC) [92]. Each locus was subsequently analyzed using Mr.Bayes [93],[94], with four Markov chains running for 2,000,000 generations (two simultaneous runs), discarding the initial 25% of the trees as burn-in. Topology and branch length priors were left at default settings. Convergence of runs was examined in a random subset of loci. Two million generations was sufficient to reach convergence in all examined cases. Each posterior distribution was used as input for a second-stage MCMC, Bayesian concordance analysis, using Bayesian Untangling of Concordance Knots (BUCKy) software [71] with 100,000 MCMC updates. The Bayesian concordance model incorporates a prior distribution of gene tree concordance (α). To examine the effects of variation in this prior, several values were compared: complete independence among loci (α  =  infinity), a high probability of concordance among loci (α = 10^−6^), and an intermediate level of concordance (α = 1). Analyses of locus size were performed for each topology by filtering out loci that did not have a posterior probability of at least 0.95, allowing unambiguous assignment of each locus to one of the three topologies. Locus size was defined with only the mouse sequences rather than including gaps introduced from the alignment with rat.

All phylogenetic analyses were repeated in a maximum likelihood (ML) framework to determine if inferences of the single-locus phylogenetic histories were robust to methodology. Each locus was allowed to follow an independent model of molecular evolution, as determined by ModelTest [95]. The highest scoring model was selected based upon Akaike's information criterion (AIC). ML searches were conducted using PAUP* version 4.0b10 [96] using 500 bootstrap replicates to assess support. Because of the small number of taxa, heuristic search settings were left at default (TBR branch swapping, stepwise starting tree, simple taxon addition, 10 replicates). Rank correlations between posterior probability from Mr.Bayes and bootstrap support from PAUP* were calculated for each topology using Spearman's rank correlation test. Concordance factors were calculated from the maximum likelihood trees by filtering out loci that did not have at least 95% bootstrap support and calculating the proportion of loci that supported a particular topology [23].

### Star Tree Paradox

Resolved gene trees can be supported by artificially high posterior probabilities when the actual tree is a hard polytomy or has a very short internal branch (the “star tree paradox”) [66],[68]. To determine whether the gene trees across the house mouse genome were resolved by a short internal branch or were actually polytomies, the maximum likelihood of the Bayesian majority rule consensus tree was calculated along with the maximum likelihood of the tree with an internal branch constrained to length zero. Both likelihood scores were calculated with PAUP*, using identical model parameters summarized from the Mr.Bayes runs. The likelihood ratio test statistic was calculated as -2(-ln(restricted model) – ln(full model)). This test statistic was assumed to fit a mixed chi-square distribution where α was twice the comparable value in a non-mixed, chi-square distribution of one degree of freedom (significance was set at α = 0.1, 1 df, χ^2^ = 2.706) [70].

### Correlation with Recombination Rate

A sliding window analysis was used to compare locus size and recombination rate across the genome. Recombination rate was estimated within 1 Mb windows by linearly regressing genetic map position [64],[65] on physical position of NCBI build 36 of the mouse genome. Windows were shifted at 250 kb intervals. The slope of this regression was used as an estimate of recombination rate (in cM/Mb) for the physical position at the midpoint of the window. If the window had less than three markers, it was discarded. The midpoint of each locus was paired with the closest estimate of recombination rate. Correlations were calculated using Spearman's rank correlation test. Statistical significance was estimated by permuting the recombination rate estimates while holding locus size constant, calculating the correlation, and repeating this permutation 100,000 times to generate a null distribution of correlation coefficients. Because the distance between a locus midpoint and the nearest estimate of recombination rate varied, correlation analyses were conducted by progressively restricting the data set from all data to only including loci within: 1 Mb, 500 kb, 2501kb, 100 kb, 75 kb, and 50 kb of a recombination rate estimate. This procedure tested whether the correlation became stronger when the analysis was restricted to loci associated with the closest estimates of recombination rates.

To ascertain whether there were statistical differences in recombination rate between loci with different sizes, the median recombination rate was determined for the 352 (2.5%) largest and smallest locus sizes. Statistical significance was estimated under the null hypothesis that the largest and smallest loci were actually from the same distribution. Both the largest and smallest loci were pooled. Random samples of loci were drawn with replacement from the combined pool to generate 352 large loci and 352 small loci test sets. The difference in median recombination rate between the two test pools was used as the test statistic. Sampling with replacement was repeated 100,000 times to generate a null distribution of test statistics.

### Long-Branch Attraction

To determine whether the phylogenetic discordance observed across gene trees was caused by long-branch attraction to one of the mouse lineages within the ingroup [72],[73], we compounded any effect of long-branch attraction by randomizing the nucleotides of the rat sequence at every locus across chromosomes 18 and 19 [97]. This increased the length of the rat branch, erasing phylogenetic signal between rat and house mice. Each randomized locus was then subjected to Bayesian phylogenetic analysis and used as input for the Bayesian concordance analysis as described above with a prior of complete independence among loci (α  =  infinity). The entire randomization test was repeated five times. A chi-square test was conducted on each replicate to determine whether the concordance factors of the three topologies differed from the 1/3, 1/3, 1/3 proportions expected if rat was randomly pairing with any of the three ingroup taxa. Only one replicate is reported, as each replicate had nearly identical results.

### Ascertainment Bias Simulation

Yang et al. [59] documented an ascertainment bias in these data against SNPs that are consistent with *a M. m. musculus/M. m. domesticus* topology. To understand the effects of this bias on our results, we conducted a series of simulations that mimicked varying degrees of bias in SNP discovery by artificially shortening the length of the *M. m. castaneus* branch. Ten percent to 80% of *M. m. castaneus* specific SNPs were randomly removed from the orthologous blocks generated by the whole-genome alignment with rat, resulting in eight separate concordance analyses with increasing levels of ascertainment bias. To remove the SNPs, a randomly selected *M. m. castaneus* genotype was changed to the corresponding C57BL/6J genotype at that position to simulate a false negative result on a high-density oligonucleotide array [57]. Each biased orthologous block was partitioned by MDL, subjected to Bayesian phylogenetic analysis, and used as input for the Bayesian concordance analysis as described above. For computational tractability, the ascertainment bias simulations and analyses were restricted to chromosomes 18 and 19, which exhibited different patterns of chromosome-wide concordance.

## Supporting Information

Figure S1 Distribution of locus sizes. Using a minimum description length principle, the genome was partitioned into 14,081 loci with a median size of 98,238 bp (SD 312,637 bp) and a maximum locus size of 7.21 Mb. Loci greater than 1 Mb in size are not shown.
(0.30 MB TIF)

Figure S2 Recombination rate within large and small loci. The 2.5% largest loci (blue) have a significantly lower recombination rate as compared to the 2.5% smallest loci (red) (*p*<0.00001), suggesting the minimum description length principle partitioned the genome in a biologically informative manner.
(0.33 MB TIF)

Figure S3 Single locus posterior probabilities. 84.9% of loci are supported by a high posterior probability (>0.9) from the single-locus Bayesian phylogenetic analyses, suggesting the minimum description length principle partitioned the genome in a phylogenetically informative manner.
(0.58 MB TIF)

Figure S4 Fine-scale phylogenetic discordance. The posterior probability of each topology is mapped throughout the genome to characterize fine-scale patterns of discordance. Position along the chromosomes is indicated on the x-axis (Mb) and the posterior probability of each topology is on the y-axis. Colors correspond to the three topologies.
(6.81 MB TIF)

Figure S5 Median locus size for each of the three topologies. Median locus size for each topology parallels the rank order of the concordance factors on both the autosomes and the X chromosome. Colors correspond to the three topologies.
(0.28 MB TIF)

Figure S6 Maximum and minimum penalties against breakpoints for the minimum description length partitioning. Both the maximum (3) and minimum (0.9039) penalties were applied to the partitioning of chromosomes 18, 19, and X. Using a minimum penalty roughly doubles the number of loci on each chromosome, but the chromosome-wide concordance factors remain similar. Colors correspond to the three topologies. Error bars are 95% credibility intervals.
(0.32 MB TIF)

Figure S7 Varied starting interval sizes for the minimum description length partitioning. A range of SNP intervals was applied to the partitioning of chromosomes 18 and 19. There are no significant differences in the concordance factors between the first three starting intervals: 25, 50, or 100 SNPs. Colors correspond to the three topologies. Error bars are 95% credibility intervals.
(0.38 MB TIF)

Figure S8 Phylogenetic discordance and long-branch attraction. The rat sequence was randomly shuffled to erase any phylogenetic signal between rat and house mice on chromosomes 18 and 19. Without the sequence shuffled (A), topologies significantly deviate from a 1/3, 1/3, 1/3 ratio. With the rat sequence shuffled (B), the topologies converge to a 1/3, 1/3, 1/3 ratio. This indicates the rat sequence provides a strong phylogenetic signal and the patterns of discordance are not driven by long-branch attraction. Colors correspond to the three topologies. Error bars are 95% credibility intervals.
(0.38 MB TIF)

Table S1 Genomic locations and posterior probabilities of the 14,081 loci (computed with a prior probability of gene tree concordance set at α = 1).
(1.29 MB XLS)
