# Supplementary figures and images for: Fine-Scale Phylogenetic Discordance across the House Mouse Genome
Source: PLoS Genet. 2009 Nov 20;5(11):e1000729. doi: 10.1371/journal.pgen.1000729 (PMC2770633; doi:10.1371/journal.pgen.1000729)

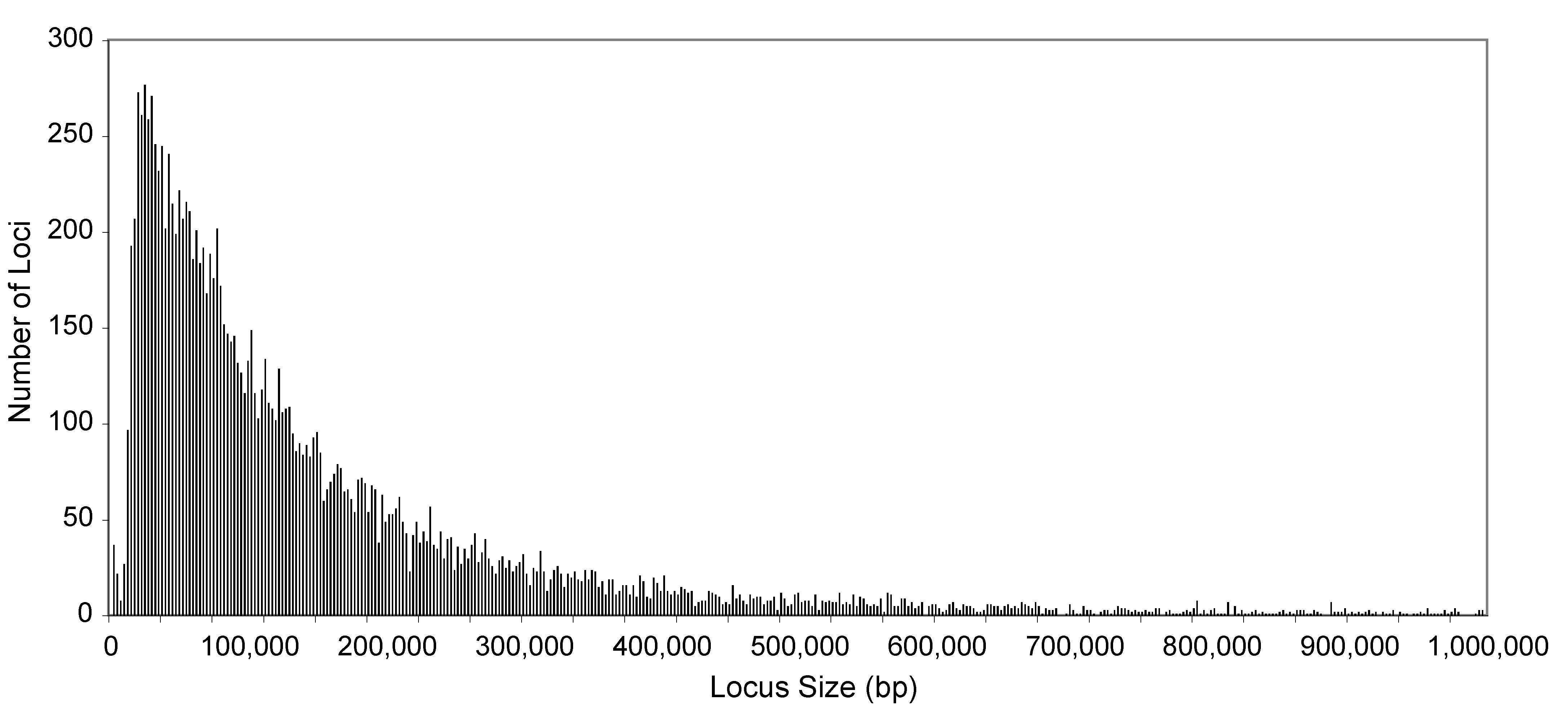

Supplement: Figure S1 — Distribution of locus sizes. Using a minimum description length principle, the genome was partitioned into 14,081 loci with a median size of 98,238 bp (SD 312,637 bp) and a maximum locus size of 7.21 Mb. Loci greater than 1 Mb in size are not shown. (0.30 MB TIF) [file pgen.1000729.s001.tif]

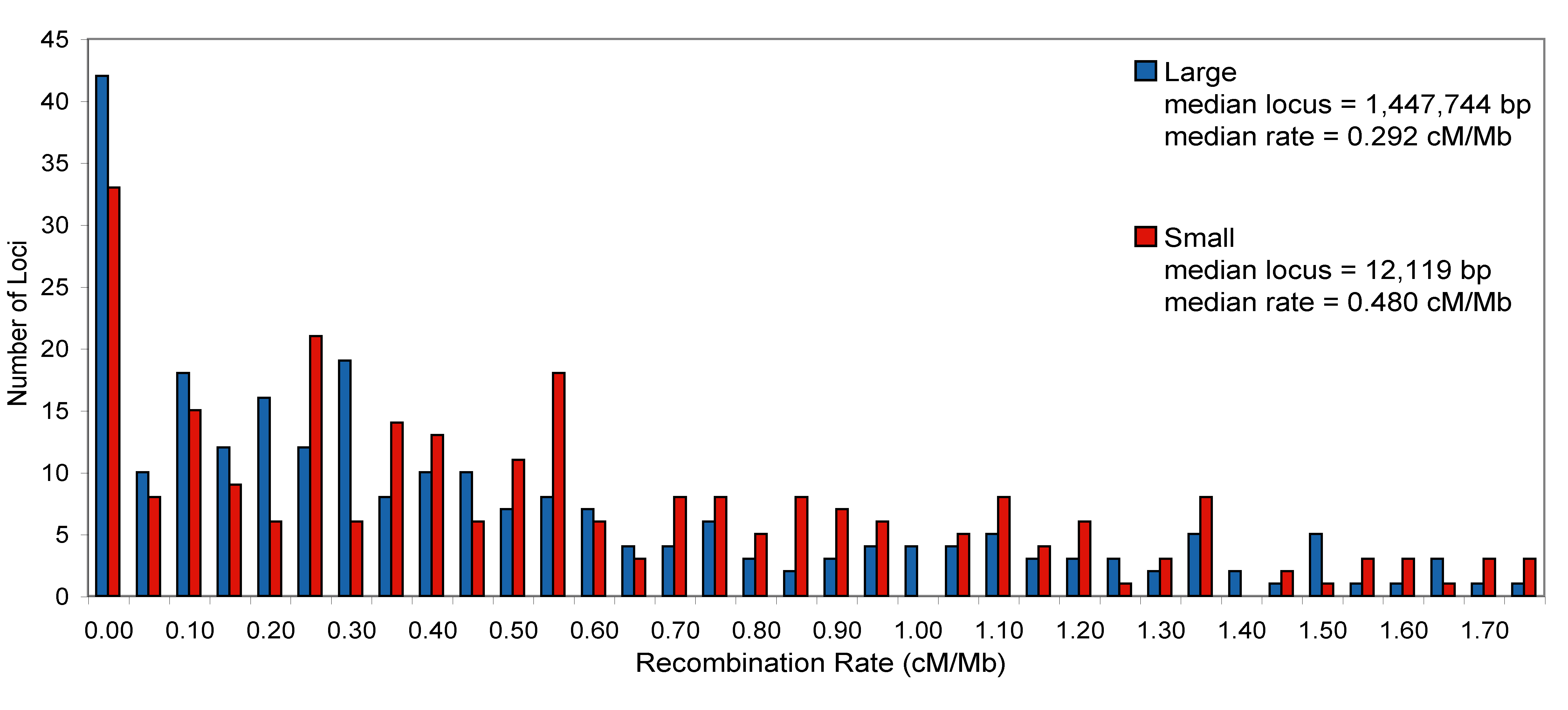

Supplement: Figure S2 — Recombination rate within large and small loci. The 2.5% largest loci (blue) have a significantly lower recombination rate as compared to the 2.5% smallest loci (red) (p<0.00001), suggesting the minimum description length principle partitioned the genome in a biologically informative manner. (0.33 MB TIF) [file pgen.1000729.s002.tif]

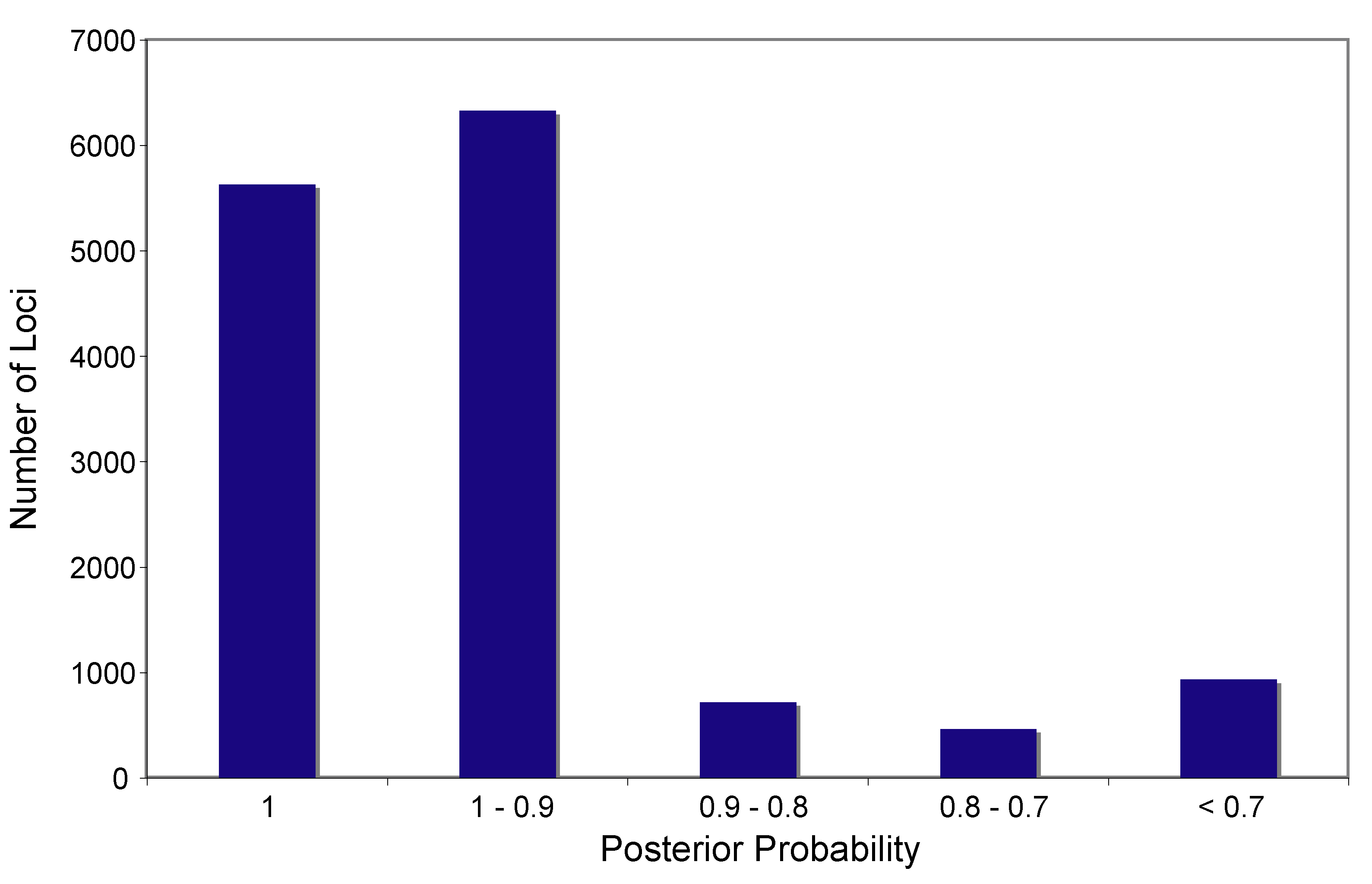

Supplement: Figure S3 — Single locus posterior probabilities. 84.9% of loci are supported by a high posterior probability (>0.9) from the single-locus Bayesian phylogenetic analyses, suggesting the minimum description length principle partitioned the genome in a phylogenetically informative manner. (0.58 MB TIF) [file pgen.1000729.s003.tif]

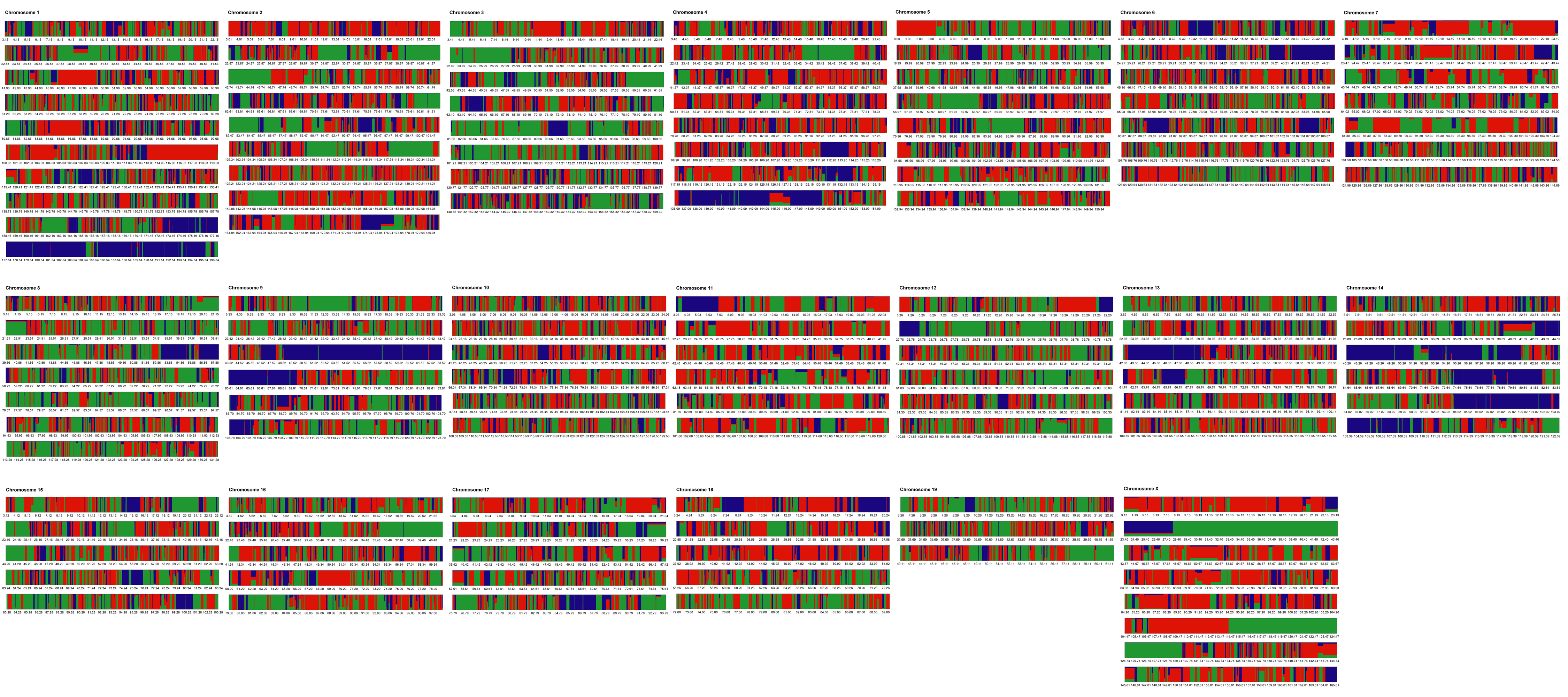

Supplement: Figure S4 — Fine-scale phylogenetic discordance. The posterior probability of each topology is mapped throughout the genome to characterize fine-scale patterns of discordance. Position along the chromosomes is indicated on the x-axis (Mb) and the posterior probability of each topology is on the y-axis. Colors correspond to the three topologies. (6.81 MB TIF) [file pgen.1000729.s004.tif]

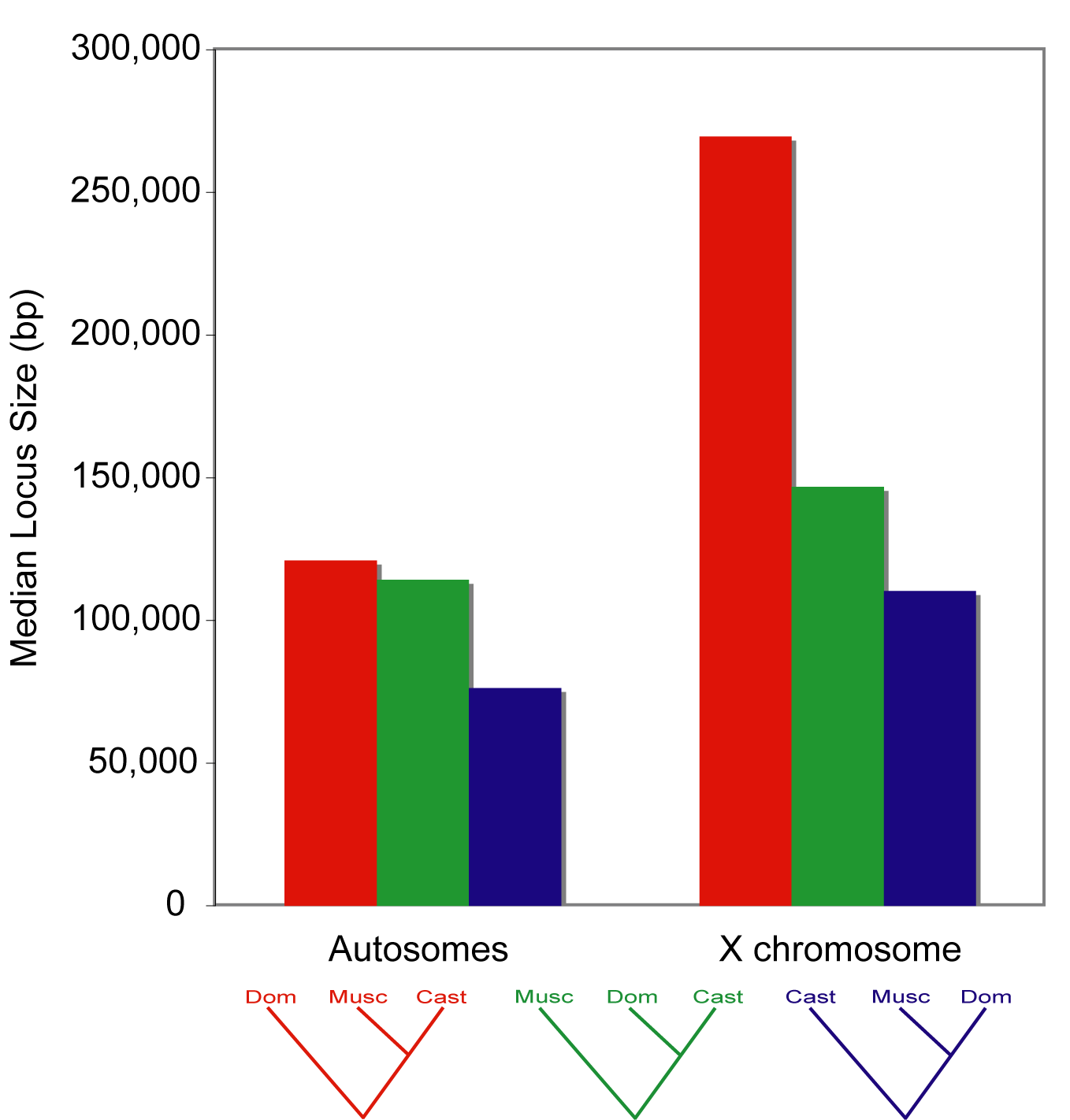

Supplement: Figure S5 — Median locus size for each of the three topologies. Median locus size for each topology parallels the rank order of the concordance factors on both the autosomes and the X chromosome. Colors correspond to the three topologies. (0.28 MB TIF) [file pgen.1000729.s005.tif]

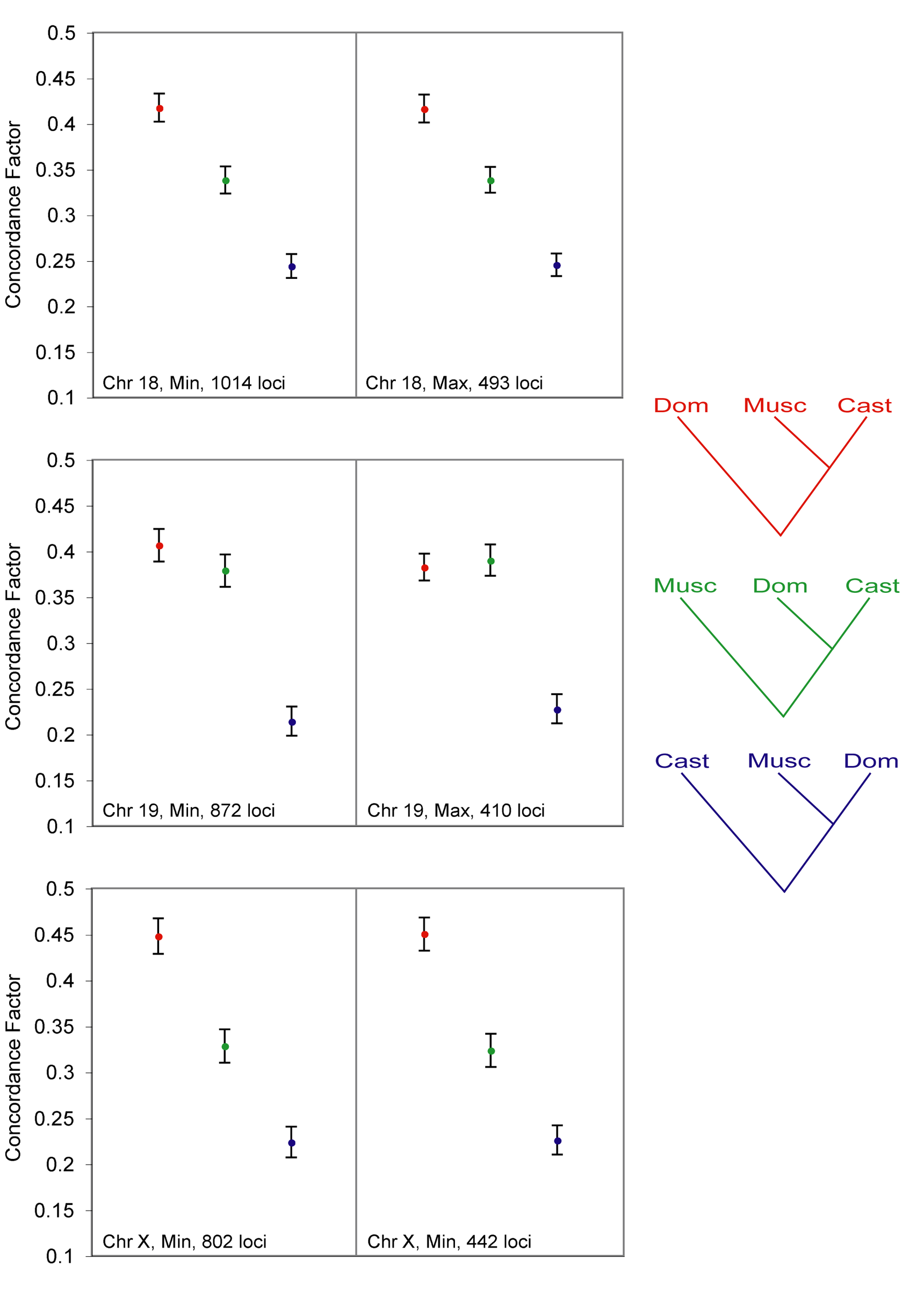

Supplement: Figure S6 — Maximum and minimum penalties against breakpoints for the minimum description length partitioning. Both the maximum (3) and minimum (0.9039) penalties were applied to the partitioning of chromosomes 18, 19, and X. Using a minimum penalty roughly doubles the number of loci on each chromosome, but the chromosome-wide concordance factors remain similar. Colors correspond to the three topologies. Error bars are 95% credibility intervals. (0.32 MB TIF) [file pgen.1000729.s006.tif]

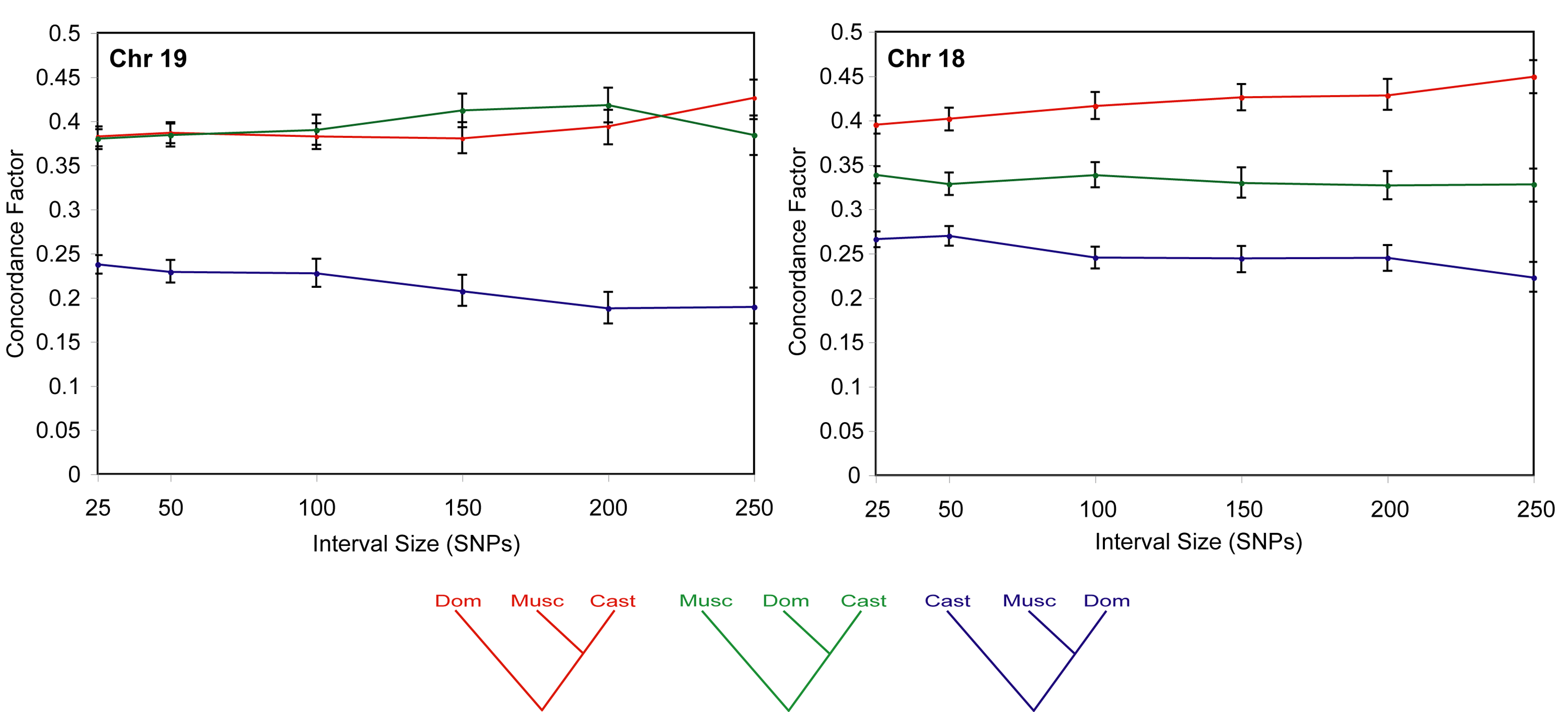

Supplement: Figure S7 — Varied starting interval sizes for the minimum description length partitioning. A range of SNP intervals was applied to the partitioning of chromosomes 18 and 19. There are no significant differences in the concordance factors between the first three starting intervals: 25, 50, or 100 SNPs. Colors correspond to the three topologies. Error bars are 95% credibility intervals. (0.38 MB TIF) [file pgen.1000729.s007.tif]

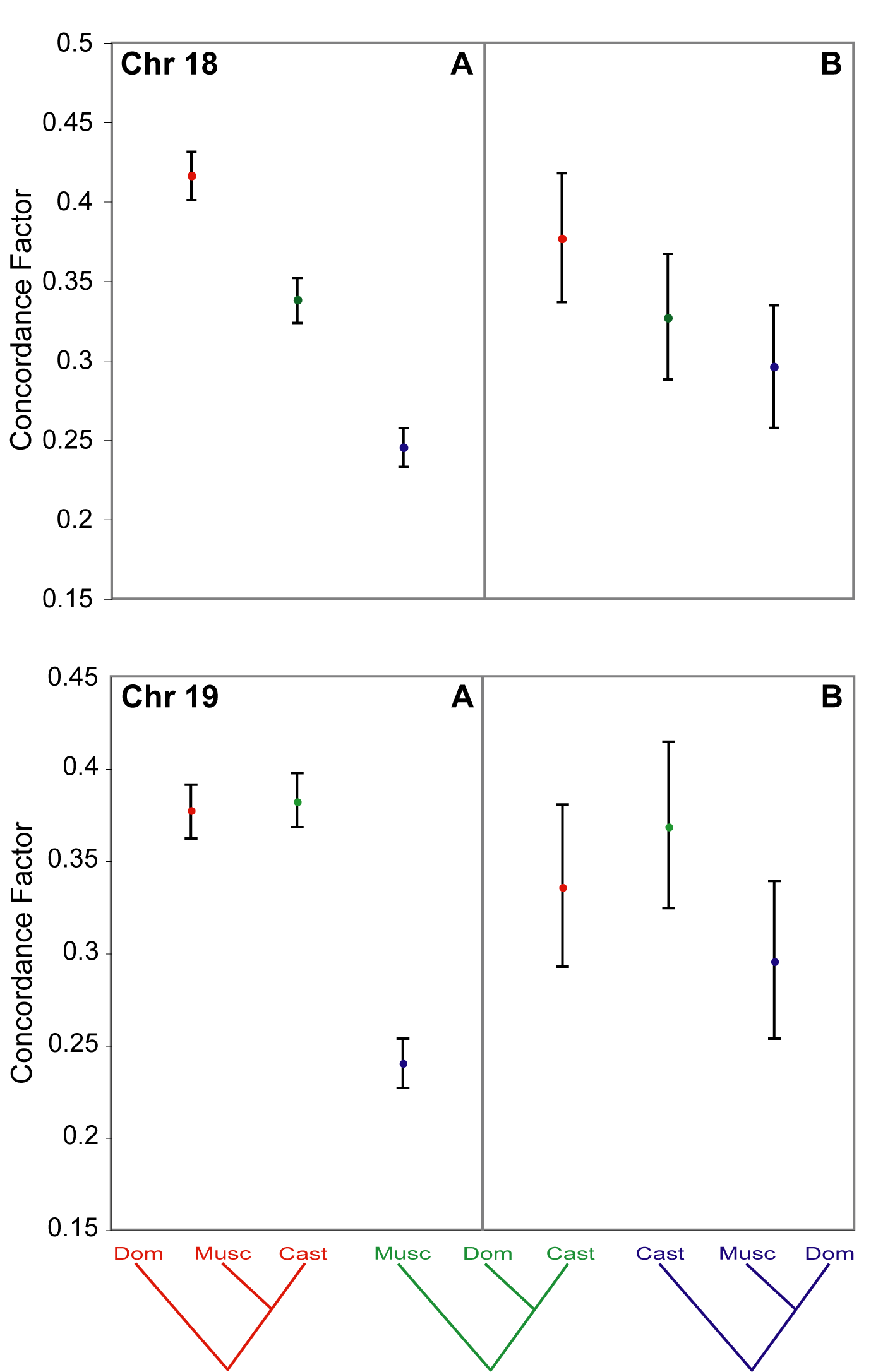

Supplement: Figure S8 — Phylogenetic discordance and long-branch attraction. The rat sequence was randomly shuffled to erase any phylogenetic signal between rat and house mice on chromosomes 18 and 19. Without the sequence shuffled (A), topologies significantly deviate from a 1/3, 1/3, 1/3 ratio. With the rat sequence shuffled (B), the topologies converge to a 1/3, 1/3, 1/3 ratio. This indicates the rat sequence provides a strong phylogenetic signal and the patterns of discordance are not driven by long-branch attraction. Colors correspond to the three topologies. Error bars are 95% credibility intervals. (0.38 MB TIF) [file pgen.1000729.s008.tif]
